# Silibinin down-regulates FAT10 and modulate TNF-α/IFN-γ-induced chromosomal instability and apoptosis sensitivity

**DOI:** 10.1242/bio.011189

**Published:** 2015-07-03

**Authors:** Yun Gao, Steven Setiawan Theng, Way-Champ Mah, Caroline G. L. Lee

**Affiliations:** 1 Division of Medical Sciences, Humphrey Oei Institute of Cancer Research, National Cancer Centre Singapore, 169610, Singapore; 2 NUS Graduate School of Integrative Sciences and Engineering, National University of Singapore, 119077, Singapore; 3 Department of Biochemistry, Yong Loo Lin School of Medicine, National University of Singapore, 119077, Singapore; 4 Duke-NUS Graduate Medical School Singapore, 169547, Singapore

**Keywords:** FAT10, Silibinin, TNF-α, IFN-γ, Chromosomal instability, Apoptosis

## Abstract

Pleiotropic pro-inflammatory cytokines, TNF-α and IFN-γ (TI), play important yet diverse roles in cell survival, proliferation, and death. Recent evidence highlights FAT10 as a downstream molecule in the pathway of inflammation-induced tumorigenesis through mediating the effect of cytokines in causing numerical CIN and protecting cells from cytokines-induced cell death. cDNA microarray analysis of cells treated with TI revealed 493 deregulated genes with FAT10 being the most up-regulated (85.7-fold) gene and NF-κB being the key nodal hub of TI-response genes. Silibinin is reported to be a powerful antioxidant and has anti-C effects against various carcinomas by affecting various signaling molecules/pathways including MAPK, NF-κB and STATs. As NF-κB signaling pathway is a major mediator of the tumor-promoting activities of TI, we thus examine the effects of silibinin on TI-induced FAT10 expression and CIN. Our data showed that silibinin inhibited expression of FAT10, TI-induced chromosome instability (CIN) as well as sensitizes cells to TI-induced apoptosis. Significantly, silibinin suppressed intra-tumorally injected TNF-α-induced tumor growth. This represents the first report associating silibinin with FAT10 and demonstrating that silibinin can modulate TI-induced CIN, apoptosis sensitivity and suppressing TNF-α-induced tumor growth.

## INTRODUCTION

First proposed by Virchow during the 19th century, a causal link between inflammation and various cancers, including liver, colorectal, gastric, lung, breast and cervical cancers is now supported by a large number of epidemiological and experimental data (Hojilla et al., 2008; Itzkowitz and Yio, 2004; McNamara and El-Omar, 2008; Szabo et al., 2007). During tumorigenesis, various cytokines are secreted either by the tumor cells itself or cells in its vicinity, to maintain a chronic pro-inflammatory and immunosuppressive condition in the tumor microenvironment and promote cell proliferation, apoptosis resistance, angiogenesis, invasion, and metastasis (Colotta et al., 2009; Lin and Karin, 2007; Peebles et al., 2007). Many cytokines modulate the NF-κB (nuclear factor kappa-B) signaling pathway leading to the pathogenesis of chronic inflammatory diseases including cancer (Yamamoto and Gaynor, 2001).

Targeting molecules downstream of the NF-κB signaling pathway might be a better choice as a therapeutic target for inflammation-associated cancers as this may have lesser side effects (Yamamoto and Gaynor, 2001). One of the downstream gene targets of TNF-α (tumor necrosis factor alpha) is FAT10 (HLA-F-adjacent transcript 10) or di-ubiquitin (UBD), whose expression is synergistically inducible by TNF-α and IFN-γ (interferon gamma) (TI) as well as retinoids (Dokmanovic et al., 2002; Lukasiak et al., 2008; Raasi et al., 1999). FAT10 is over-expressed in inflammation-associated cancers including hepatocellular carcinoma (HCC) and gastrointestinal cancers (Lee et al., 2003; Lukasiak et al., 2008), non-small cell lung cancer (Heighway et al., 2002) as well as glioma (Yuan et al., 2012). Our recent study also provides evidence that FAT10 is one of the downstream molecules in the pathway of inflammation-induced tumorigenesis (Gao et al., 2014) mediating the effect of TNF-α in causing numerical chromosome instability (CIN) (Gao et al., 2014; Ren et al., 2006) through its interaction with the mitotic checkpoint protein MAD2 (mitotic arrest deficient-2) (Liu et al., 1999; Theng et al., 2014) and protecting cells from TNF-α-induced cell death (Ren et al., 2011). FAT10 is an 18 kDa protein belonging to the ubiquitin-like modifier (UBL) family of proteins. It comprises two tandem head-to-tail ubiquitin-like domains with 29% and 36% identity to ubiquitin, respectively, that is separated by a short linker (Liu et al., 1999). Similar to ubiquitin, FAT10 contains a C-terminal di-glycine motif which is important for conjugation to different substrates including p53 (Li et al., 2011; Raasi et al., 2001). FAT10 was reported to be activated by the E1-enzyme UBA6/E1-L2 (Chiu et al., 2007) and USE1 (Aichem et al., 2010).

Silibinin, a flavanone in milk thistle (*Silybum marianum* L.), is widely used to treat a range of liver and gallbladder disorders, including hepatitis, cirrhosis, and as a hepatoprotectant against poisoning from wild mushroom, alcohol, chemical, and environmental toxins (Loguercio and Festi, 2011; Rainone, 2005). Over the last 15 years, silibinin has been shown to exert anticancer and cancer chemopreventive effects by affecting various signaling molecules/pathways involved in malignant cell growth (Tsuda et al., 2004). Silibinin was reported to alter MAPK, NF-κB and STATs signaling as well as cell cycle regulators (Cheung et al., 2010). Additional studies showed that silibinin also inhibits TI-induced STATs, MAPKs, NF-κB and AP-1 activation (Chittezhath et al., 2008).

Here, we report the first investigation to our knowledge of the role of silibinin in modulating TI-induced, FAT10-associated properties including CIN, apoptosis and tumor growth.

## RESULTS

### Upon TNF-α/IFN-γ treatment, differentially expressed genes centered on NF-κB pathway

**Fig. 1.**
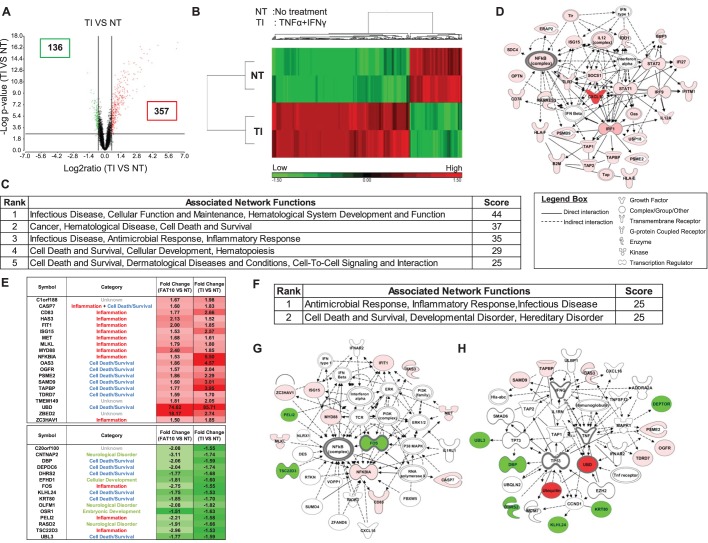
**Differentially expressed genes and pathways deregulated in HCT116 cells treated with TNF-α/IFN-γ (TI).** (A) Volcano plot and (B) Heat map of expression profiles of genes deregulated in cells treated with TI compared with untreated cells (NT). Red denotes high expression; green indicates low expression. (C) Top 5 networks of genes that were differentially expressed in cells treated with TI compared to NT. (D) Schematic representation of the top ranked network (infectious disease, cellular function and maintenance) of these differentially expressed genes predicted by the ingenuity pathway analysis (IPA). The types of molecules are annotated in the legend box. Genes linked to at least three other genes in the network are referred to as hub genes. (E) Genes commonly differentially expressed in FAT10-expressing cells and cells treated with TI. Down-regulated genes (relative to untreated cells) are represented in green; up-regulated genes (relative to untreated cells) are represented in red. The intensity of the colors represent the strength of the gene deregulation. (F) Top 2 networks of commonly differentially expressed genes in FAT10- and TI treated cells as predicted by IPA. Red denotes up-regulated genes while green represents down-regulated genes. Schematic representation of the (G) top ranked network (antimicrobial response, inflammatory response, infectious disease) and (H) second top ranked network (cell death and survival, developmental disorder, hereditary disorder) of these commonly differentially expressed genes in FAT10- and TI treated cells as predicted by IPA.

Transcriptome profiling of HCT116 cells treated with TI for 8 h revealed 493 differentially expressed genes with 357 up-regulated and 136 down-regulated genes (Fig. 1A,B and supplementary material Table S1). As expected, the top 5 networks as revealed by ingenuity pathway analyses (IPA) are primarily involved in infectious disease, inflammatory response, cancer, cell-death and survival (Fig. 1C) with key molecules of the top network centered primarily on key pro-inflammatory molecules including NF-κB (Fig. 1D). As FAT10 was clearly the most highly up-regulated gene (85.7 fold) in this transcriptome profile (supplementary material Table S1), we proceeded to identify the common genes and pathways from cells treated with TI and cells with over-expression of the FAT10 gene by comparing this transcriptome profile with the transcriptome profile of HCT116 cells with over-expression of the FAT10 gene. As evident in Fig. 1E, a total of 35 (20 up-regulated and 15 down-regulated) genes were identified to be commonly differentially expressed in TI and FAT10-expressing cells. More than 70% of these genes are in the anti-microbial/inflammatory or cell-death/survival pathways (Fig. 1E). In fact, the top 2 networks with equally strong prediction scores computed by IPA are antimicrobial/inflammatory response/infectious disease and cell-death and survival (Fig. 1F). The key molecules in these pathways revolved around molecules involved in inflammation (e.g. TNF, NF-κB and IFN) and cell-death/survival (e.g. FOS, p53) suggesting FAT10 play roles in inflammation as well as cell-death/survival through TNF and NF-κB (Fig. 1G,H) which concurs with our previous observations (Ren et al., 2011).

### Silibinin likely target NF-κB pathway to revert gene expression deregulated by TNF-α/IFN-γ to normal

**Fig. 2.**
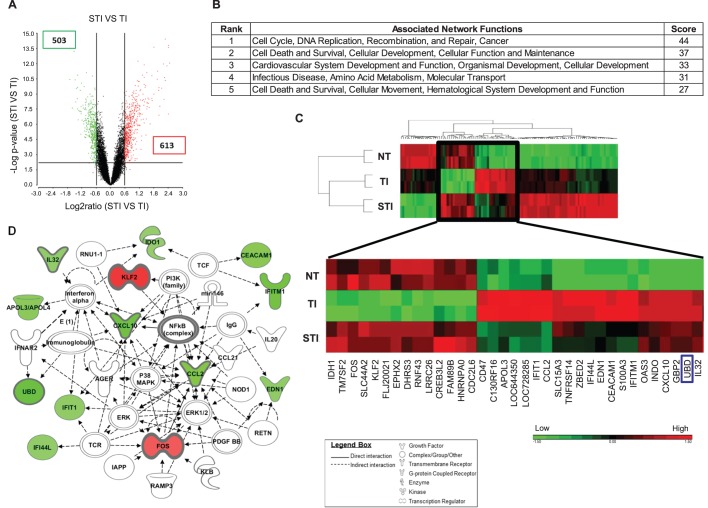
**Differentially expressed genes and pathways deregulated in HCT116 cells treated with silibinin/TNF-α/IFN-γ (STI) compared to cells treated with TNF-α/IFN-γ (TI).** (A) Volcano plot of expression profiles of genes deregulated in STI versus TI cells. (B) Table shows the top 5 networks of genes differentially expressed in STI versus TI cells. (C) Heat map of expression profiles of genes deregulated in STI versus TI treated cells. Subset of genes treated with STI that facilitate the reversion of expression profiles of cells from TI treated (TI) to untreated (NT) levels are magnified below the heat map. UBD gene (FAT10) is boxed in blue. Red indicates high expression; green indicates low expression. (D) Schematic diagram that shows the top ranked network (cell-to-cell signaling and interaction, cellular movement, connective tissue development and function) of this subset of genes that can revert TI to NT phenotype.

To elucidate the effect of silibinin on TI-treated cells, cDNA microarray analyses were performed on cells treated with silibinin/TI (STI) versus cells treated with TI. Fig. 2A (supplementary material Table S2) shows a total of 1116 differentially expressed genes, 503 of which are down-regulated and 613 up-regulated. The top associated network function affected by silibinin was found to be cell-cycle, DNA replication, recombination and repair and cancer (Fig. 2B). A subset of 35 genes, which included FAT10 (UBD) (boxed in blue), was found to facilitate the reversion of expression profiles of cells from TI treated to un-treated (NT) levels (Fig. 2C, supplementary material Table S3). The top network with the strongest prediction scores computed from IPA for this subset of 35 genes was cell-to-cell signaling and the nodal molecule in this network remains as NF-κB complex with several key deregulated molecules which includes FAT10 (UBD), FOS, KLF2 and chemokines, CXCL10, CCL2 and IL32. Hence, silibinin likely target NF-κB pathway to revert gene expression deregulated by TNF-α/IFN-γ to normal (Fig. 2D, supplementary material Table S4).

### TNF-α/IFN-γ induce endogenous FAT10 expression through NF-κB while silibinin inhibits TNF-α/IFN-γ-induced FAT10 gene expression transcriptionally through down-regulating IκBα and disrupting TNF-α/IFN-γ stimulated p65 nuclear translocation and NF-κB activation

**Fig. 3.**
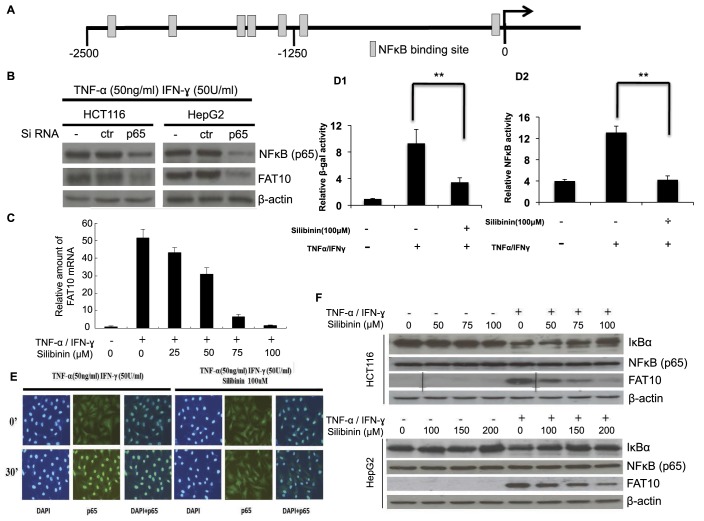
**Silibinin inhibits TNF-α/IFN-γ (TI)-induced endogenous FAT10 expression through NF-κB signaling pathway.** (A) Seven predicted binding sites of NF-κB were found within 2.5 kb upstream of FAT10 promoter region by GenomatixMatInspector Version 8.0 (http://www.genomatix.de). (B) p65 plays a role in modulating FAT10 expression induced by TI. Cells were electroporated with control siRNA (ctr) or siRNAs against p65 (p65) and grown for 24 h. They were then cultured with or without TI for 8 h before harvest. Inhibition of NF-κB subunit p65 expression by its specific siRNA and FAT10 expression were confirmed by western blot. (C) Silibinin inhibits TI-induced FAT10 mRNA levels in a dose dependent manner. Real-time reverse transcription (RT)-PCR was performed to measure the levels of FAT10 mRNA. (D1) Silibinin inhibits TI-induced FAT10 promoter and (D2) Silibinin inhibits NF-κB activities. FAT10-promoter driven β-galactosidase reporter and the NF-κB-SEAP constructs were co-transfected into HepG2 cells using Lipofectamine 2000. 36 h after transfection, cells were treated with TNF-α (50 ng/ml) and IFN-γ (50 U/ml), in the presence or absence of 100 µM silibinin. Nine hours later, cells were harvested and FAT10-promoter driven β-galactosidase reporter and NF-κB driven SEAP activities were determined and normalized against GFP expression and total protein content. All data are shown as mean±s.e. (standard error). **P<0.01 compared to no Silibinin treatment. (E) Silibinin inhibits TI-induced p65 nuclear translocation. Nuclear localization of the p65 subunit of NF-κB in HepG2 cells was detected by immunofluorescence analysis using p65 antibody. (F) Silibinin modulates key molecules of the NF-κB pathway. Western blot analyses were performed to determine the protein expression of FAT10 and NF-κB (p65) in cells treated with TI in the presence or absence of silibinin.

Our recent study suggests that FAT10 may play an important role in inflammation-induced tumorigenesis through mediating the effect of TNF-α in causing numerical CIN and protecting cells from TNF-α-induced cell death (Ren et al., 2011). Since IPA results as described above also suggest that NF-κB feature prominently in cells treated with TI and silibinin likely targets the NF-κB pathway to revert gene expression deregulated by TI to normal (Fig. 2C), GenomatixMatInspector v8.0 (http://www.genomatix.de) was employed to identify putative transcription factor binding sites associated with TI. Seven potential NF-κB binding sites were identified at the FAT10 promoter (−2500/+209) (Fig. 3A).

To evaluate the role of NF-κB in regulating the expression of FAT10, siRNAs against p65 subunit (sip65) of the NF-κB complex were used to inactivate the NF-κB pathway. As evident in Fig. 3B, inhibition of the p65 subunit of NFκB significantly attenuated the TI induction of FAT10 expression in both HCT116 and HepG2 cells suggesting that TI modulated FAT10 expression through NF-κB pathway.

As silibinin was reported to inhibit TI-induced NF-κB pathway (Chittezhath et al., 2008), and TI-associated FAT10 expression is modulated through NF-κB pathway, we would like to investigate if silibinin modulate TI-induced FAT10 expression through NF-κB pathway. Fig. 3C shows that while TI induced the transcript expression of FAT10, silibinin attenuated this induction in a dose dependent manner. To determine if the modulation of FAT10 transcript expression by silibinin is at the promoter level and through the NF-κB pathway, FAT10 promoter driven β-galactosidase reporter vector and the NF-κB-SEAP vector were co-transfected into HepG2 cells. The NF-κB-SEAP vector contains the SEAP reporter gene and is designed to monitor the activation of NF-κB. Compared to vehicle control, the increase in FAT10 promoter driven β-galactosidase activity and NF-κB driven SEAP activity were 9.55-fold and 3.15-fold, respectively, after treatment with TI for 12 h (Fig. 3D). When both TI as well as 100 µM silibinin was added simultaneously, both FAT10 promoter driven β-galactosidase activity and NF-κB activity were greatly reduced to 3.45-fold and 1.09-fold, respectively (Fig. 3D). We next performed immunofluorescence analysis of cells treated either with TI or silibinin/TI using NF-κB (p65) antibody to determine the localization of NF-κB (p65). Fig. 3E shows that upon TI induction, in the absence of silibinin, NF-κB (p65) was localized to the nucleus of the cells (Fig. 3E, left panels). In the presence of silibinin and TI, NF-κB (p65) is no longer localized to the nucleus (Fig. 3E, right panels). Taken together, these data suggest that silibinin inhibits TI-induced FAT10 transcript expression at the promoter level through inhibiting NF-κB activation and disrupting TI-induced nuclear localization of NF-κB (p65).

As degradation of IκB was reported to be essential for the nuclear translocation of NF-κB (Baeuerle and Baltimore, 1988), we next determined if silibinin affects the expression of IκBα protein. Western blot analyses of HCT116 and HepG2 cells revealed that in the absence of silibinin, upon TI treatment, total IκBα protein expression was significantly decreased, likely due to the rapid degradation of the IκBα protein (Fig. 3F). Treatment of these TI-induced cells with varying concentrations of silibinin (0–100 μM in HCT116 and 0–200 μM in HepG2 cells) for 12 h resulted in a significant dose-dependent increase in the levels of total IκBα in both cell types. The expression levels of the NF-κB (p65) remain unchanged in both these TI-induced cells treated with various concentrations of silibinin (Fig. 3F). These results suggest that silibinin may inhibit the degradation of IκBα in TI-induced cells.

### Silibinin blocks TNF-α/IFN-γ-induced chromosomal instability, and sensitizes HCT116/HepG2 cells to TNF-α/IFN-γ-induced apoptosis

**Fig. 4.**
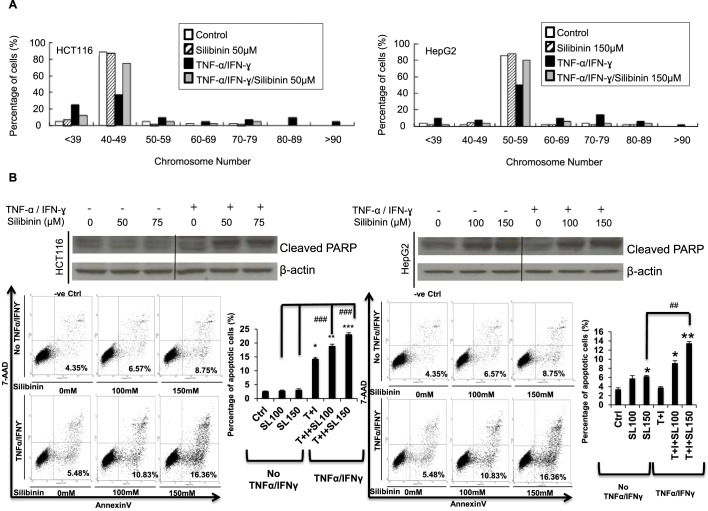
**Silibinin prevents TNF-α/IFN-γ (TI)-induced chromosomal instability (CIN) and sensitizes cells to TNF-α/IFN-γ (TI)-induced apoptosis.** (A) HCT116 (left panel) and HepG2 (right panel) cells were grown in media containing TI or silibinin/TNF-α/IFN-γ for ten passages before karyotyping was performed. 45–55 cells were karyotyped. (B) The apoptosis profile of HCT116 (left panel) or HepG2 (right panel) was determined by FACS analyses using Annexin V-FITC apoptosis detection kit and by determining cleaved-PARP with western blot. All data shown are mean±s.e. ** denotes *P*<0.01, ***denotes *P*<0.001 compared with control cells; ^#^denotes *P*<0.05, ^##^ denotes *P*<0.01 compared with silibinin treated cells only.

The functional consequences of silibinin to cells whose FAT10 expression is induced by TI were then investigated. Since FAT10 was previously reported to directly mediate the induction of chromosome instability (CIN) by TNF-α (Ren et al., 2011), we examined if silibinin can mitigate CIN in TI treated cells as silibinin inhibits FAT10 expression. As described previously, HCT116 and HepG2 cells were chronically treated with TI for about ten passages and then harvested for karyotype analysis. Greater than 80% of untreated HCT116 and HepG2 cells showed the modal chromosome number of 40–49 and 50–59, respectively (Fig. 4A). Treatment of the cells with silibinin alone did not significantly affect the modal chromosome number of the cells (Fig. 4A). Most of the TI chronically treated cells exhibited abnormal chromosome number (Fig. 4A), with only ∼40% of cells retaining normal chromosome number. Upon addition of silibinin to TI chronically treated cells, majority (∼75%) of the TI treated cells reverted to their untreated modal chromosome numbers.

**Fig. 5.**
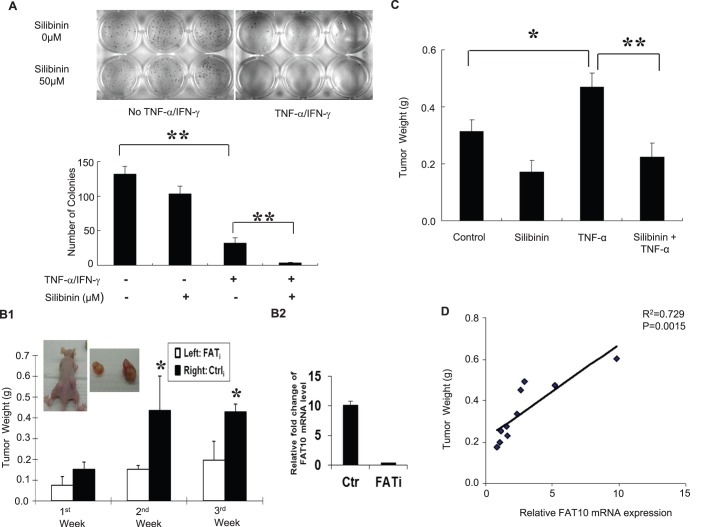
**Silibinin inhibits TNF-α-induced colony formation and tumor growth.** (A) Silibinin attenuates soft-agar colony formation of cells treated with TNF-α/IFN-γ (TI). Approximately 5000 HCT116 cells were seeded in soft agar and grown with TI or silibinin/TNF-α/IFN-γ (STI). Three weeks later, the colonies were stained for visualization. (B1) Inhibiting FAT10 expression with shRNA against FAT10 attenuates tumor growth. 5 μg/kg of TNF-α was administered intratumorally once after control (Ctrl_i_) or FAT10 (FAT_i_) shRNA containing HCT116 cells were inoculated subcutaneously into 6-week-old male athymic nude mice. Four weeks after injection, mice were sacrificed and the weights of their primary tumors were measured. (B2) Comparison of FAT10 mRNA level in FATi stable cells or control HCT116 cells injected to the mice. (C) Silibinin inhibits tumor growth. Mice with subcutaneously injected HCT116 cells and intratumorally administered TNF-α were either gavaged with saline or 100 mg/kg silibinin 5 days a week and tumor weight was determined after 7 weeks. All data in the graphs are shown as mean±s.e. *P<0.05; **P<0.01. (D) Relationship between tumor size and FAT10 expression was measured using Pearson's method.

In addition to CIN, FAT10 was also found to facilitate the resistance of cells against TNF-α induced cell death (Ren et al., 2011). As numerical CIN was also reported to facilitate the generation of more aggressive tumor cells with a reduced propensity to undergo apoptosis (Castedo et al., 2006), we thus examined if silibinin is able to sensitize these cells to TI-induced apoptosis. As evident from Annexin V-PI staining observed by FACS analyses in Fig. 4B, in the absence of TI (Untreated), silibinin did not significantly affect the apoptotic profiles of HCT116. Upon TI treatment in the absence of silibinin greater apoptosis 3.98% to 14.99% (Fig. 4B, left lower panels) was observed in HCT116 but not HepG2 (4.35% to 5.48%, Fig. 4B, right lower panels) cells. Treatment with silibinin and TI resulted in significant dose-dependent increase in apoptosis in both HCT116 (from 14.99% to 23.27%) and HepG2 (from 5.48% to 16.36%) cells (Fig. 4B, lower panels). Consistently, significantly higher levels of cleaved PARP was observed in cells treated with silibinin and TI, which suggests that silibinin sensitizes HepG2 and HCT116 cells to TI-induced apoptosis. Similarly, significantly less colonies on soft-agar was observed in HCT116 cells treated with silibinin and TI compared to those treated with only TI (Fig. 5A).

### Silibinin inhibits TNF-α induced tumor growth

Due to the toxicity of the combination TI in mice, we thus focused on the role of silibinin in modulating only TNF-α induced tumor growth through FAT10. We first evaluated the role of FAT10 in modulating TNF-α induced tumor growth. HCT116 cells stably expressing either FAT10 siRNA (FAT_i_) or control siRNA (Ctrl_i_) were inoculated subcutaneously into athymic nude mice and low dose of TNF-α (5 μg/kg) was administered intra-tumorally once, a week after tumor graft. As shown in Fig. 5B, after TNF-α treatment, significantly smaller tumors were observed on the left side of the mice which was inoculated with FAT10 siRNA (FAT_i_) expressing cells compared to the right flank which was injected with control siRNA (Ctrl_i_) suggesting that the FAT10 gene augmented tumor growth. We then determined if silibinin can modulate tumor growth by gavaging the mice with 100 mg/kg dose of silibinin for 5 days a week for 7 weeks after tumor graft and TNF-α administration. As evident in Fig. 5C, TNF-α treatment augmented tumor growth and silibinin administration, significantly decreased the size of tumor compared to either untreated HCT116 cells or HCT116 whose FAT10 expression is induced with TNF-α. The relationship between tumor size and FAT10 expression was examined to further evaluate the role of FAT10 in modulating tumor growth. Fig. 5D shows significant correlation (R^2^=0.7292, *P=*0.0015) between the FAT10 transcript expression and the tumor weight. These data suggest that silibinin can attenuate tumor growth partly through down-regulating FAT10 expression.

## DISCUSSION

Cytokines are one of the major mediators of inflammation that plays important yet diverse roles in cell survival, proliferation, differentiation and death. Our previous study revealed that FAT10 expression is induced by TNF-α through the NF-κB pathway in cancer cells (including HCT116 and HepG2) making them resistant to TNF-α-induced apoptosis and facilitating CIN (Ren et al., 2011). Silibinin is a flavonoid isolated from milk thistle seeds with anti-cancer effects against a variety of cancers, including human prostate adenocarcinoma, non-small human lung carcinoma and human colon cancer. Silibinin exhibits negative effects on TI-induced Stat3, Stat1, ERK1/2, and NF-κB activation (Tyagi et al., 2012). As NF-κB signaling pathway is a major mediator of the tumor-promoting activities of TNF-α, we thus present the first report of the investigation of the role of silibinin in modulating TI-induced FAT10 expression and its functional consequences.

Genes that may play important role in modulating the effects of TI were first identified through genome-wide expression analysis and 493 genes were found to be significantly deregulated upon TI treatment (Fig. 1A,B). In concordance with our understanding of the role of TI, these deregulated genes were found to belong primarily in the infectious disease, inflammatory response, cell-death and survival as well as cancer pathways (Fig. 1C). Interestingly, all the modulated molecules in the top network were up-regulated and the key molecules of this top network centered primarily on the key pro-inflammatory nodal molecule NF-κB (Fig. 1D). Thirty-five of these deregulated genes were found to be similarly deregulated in FAT10-overexpressing cells suggesting that TI treated and FAT10-overexpressing cells share common pathways represented by these 35 genes (Fig. 1E). IPA results again revealed infectious disease, antimicrobial/inflammatory response, cell-death and survival as the top 2 networks modulated by these 35 genes (Fig. 1F) with NF-κB and TNF-α representing the major key nodal molecules in these networks (Fig. 1G,H).

NF-κB has been implicated in carcinogenesis because of its critical roles in cell survival, cell adhesion, inflammation, differentiation, and cell growth (Sethi et al., 2008). NF-κB is an essential component of the TNF-α proliferation pathway and that TNF-α induced changes in IL-6, Stat3 and c-myc mRNA are dependent on NF-κB activation. Hence, the inhibition of NF-κB activation should be an effective approach for inflammation-associated tumorigenensis intervention. Interestingly, endogenous FAT10-expression can be induced by TI through the NF-κB pathway (Fig. 3B) (Ren et al., 2011) and several predicted binding sites for NF-κB were observed at the FAT10-promoter (Fig. 3A) (Canaan et al., 2006; Zhang et al., 2006). FAT10 can also activate NF-κB (Gong et al., 2010) suggesting a positive feedback-loop in which TI induces the expression of FAT10 through the NF-κB pathway and FAT10, in turn, activates NF-κB.

Hence, targeting NF-κB may be a rational approach for intervention of inflammation-associated cancers as blocking the TNF-α-NF-κB-FAT10 feedback-loop may result in less side effects. One potentially promising candidate that modulates the NF-κB pathway is silibinin, a phytochemical used as dietary supplement as an anti-hepatotoxic agent. Notably, silibinin is largely non-toxic as even high doses are tolerated by animals (2000 mg/kg/day dose by oral gavage in mice) and human (5.0 g/day, orally given as silybin-phytosome) (Agarwal et al., 2003; Flaig et al., 2007).

In this study, silibinin was introduced to HCT116 (≤100 μM) and HepG2 (≤200 μM) cells at reasonably low dose and the effect of silibinin on global gene expression profiles as well as FAT10 expression and subsequent functional effects were examined. Silibinin was found to inhibit TI-induced FAT10 promoter activity, NFκB activity, and nuclear localization as well as degradation of IκBα (Fig. 3D-F). The addition of silibinin was also found to revert the chromosome numbers of TI treated cells to numbers similar to untreated cells as well as restore and sensitize these cells to TI-induced apoptosis. Notably, *in vivo* tumor xenograft study revealed that silibinin was also able to reduce the size of the tumors formed by HCT116 cells alone or those treated with TNF-α (Fig. 5C).

In summary, our study showed that silibinin can modulate TI-induced FAT10 expression. Silibinin also restores normal chromosome numbers to cells treated with TI, sensitize these cells to TI-induced cell death and attenuates the tumor size. These data suggests that silibinin has potential application to alter the outcome of inflammation association cancers and raises the exciting possibility of using silibinin in the treatment of inflammation association cancers. Nonetheless, additional studies employing *in vivo* models are needed to substantiate this possibility.

## MATERIALS AND METHODS

### Cell line and reagents

All cell lines were purchased from American Type Culture Collection (ATCC, USA) and cultured under the recommended conditions. Transfection of plasmids was performed using Lipofectamine 2000 (Invitrogen, USA) while siRNAs were transfected using siPORT transfection reagent (Applied Biosystems, USA) or electroporated with the BTX ECM830 (BTX Instrument, USA). Rabbit anti-FAT10 polyclonal antibodies were generated as previously described (Lee et al., 2003). Anti-NF-κB (p65), anti-IκBα, anti-β-Actin antibodies and p65 siRNA were purchased from Santa Cruz Biotech (Santa Cruz, USA). Recombinant human TNF-α and IFN-γ were purchased from Invitrogen (USA) and Roche Life Science (USA), respectively.

### Quantitative real-time RT-PCR assay

Reverse transcription and quantitative real time PCR were carried out as previously described (Gao et al., 2014). The primers used are as follows: FAT10 (F: 5′-CAATGCTTCCTGCCTCTGTG, R: 5′-TGCCTCTTTG-CCTCATCACC); GAPDH (F: 5′-GACCTATGGAAACTACTTCCT, R: 5′-GTACGTGCAAGTCACAGACT).

### Colony formation in soft agar

Anchorage-independent growth of HCT116 and HepG2 cells were determined by colony formation in soft agar according to previously described protocol (Theng et al., 2014). The assays were performed in triplicate and repeated three times.

### Apoptosis analysis

To determine the apoptosis profile, cells were stained using the Annexin V-PE apoptosis kit (BD Biosciences, USA). Cells were grown to 70% confluency before treatment with various concentrations of TI or silibinin/TI for 12 h. The cells were then stained at room temperature in the dark for 15 min in 200 µl of buffer containing Annexin V-PE and 7-AAD. FACS buffer was added, and cells were analyzed immediately by FACSCalibur flow cytometer (BD Biosciences).

### Cytogenetic analysis

Cells at 10% confluency were grown in TI or silibinin/TI supplemented medium for 24 h before treatment was removed and the cells were allowed to grow until confluence. The cells were then passaged and the attached passaged cells were then again treated with TI or silibinin/TI for another 24 h. This process was repeated for about ten passages. The cells were then harvested for determination of chromosome number. The analyses of chromosome numbers were performed as previously described (Ren et al., 2006).

### Luciferase reporter assay

2.5 kb fragment upstream the transcriptional start site of FAT10 was cloned into a reporter gene (β-galactosidase) expression vector as previously mention in our previous study (Zhang et al., 2006). This FAT10-promoter reporter and NF-κB-SEAP vector (Clontech, USA) were cotransfected into HepG2 cells. Thirty-six hours after transfection, 50 ng/ml of TNF-α, and 50 U/ml of IFN-γ were introduced to the cells. Cells were then harvested 6 to 9 h later. FAT10 promoter driven β-galactosidase activity and NF-κB driven secreted alkaline phosphatase (SEAP) activity were normalized against the GFP expression and total protein amount. β-galactosidase activity was assayed using chlorophenol red-b-D-galactopyranoside as substrate in a kinetic assay at 570 nm with a SpectraMAX^PLUS^ (Molecular Devices, USA), whereas EGFP fluorescence was quantitated at 509 nm after excitation at 488 nm.

### NF-κB SEAP reporter assay

Activation of the NFκB signal transduction pathway was determined by measuring the SEAP (secreted alkaline phosphatase) from the NF-κB-SEAP vector. Cells were co-transfected with the pNF-κB-SEAP and GFP construct. 36 h after transfection, supernatants were collected and the Great EscAPe SEAP Kit 2.0 (Clontech) was used for SEAP detection. SEAP signal was read with Fluoroskan Fluorometer (Thermo Scientific, USA). NF-κB transcriptional activity was normalized to GFP expression level to correct for variability in transfection efficiency.

### Tumor xenograft study

HCT116 cells were prepared as single-cell suspensions in sterile PBS at a concentration of 2×10^7^ cells/ml, and a volume of 250 µl (5×10^6^ cells) was injected subcutaneously into 6-week-old male athymic nude mice (BALB/c nu/nu; purchased from Seiken Co. Ltd., Singapore). The healthy animals were randomly divided in to four groups after one week of implantation. One group (*n*=8 mice) was gavaged with 200 μl of saline/mouse/day while the other group (*n*=8) was gavaged with 100 mg/kg dose of silibinin in 200 μl of saline/mouse/day for 5 days a week. Animals were monitored for tumor growth (by palpation), general health, body weight and diet consumption twice weekly throughout the study which was terminated after 7 weeks of the treatment. This study was performed according to the guidelines and with the approval of the SingHealth Institutional Animal Care and Use Committee (IACUC) (Protocol number: 2009/SHS/504).

### Microarray analysis and statistics

Microarray data was analyzed using the Partek Genomics Suite Software version 6.6 (Partek Inc., USA). Genes with significantly differential expression were determined based on ANOVA. Two-tailed *t*-test was utilized to evaluate the significance of the difference.

### Immunofluorescence analysis

HCT116 cells were grown on coverslips and fixed in 4% paraformaldehyde solution. These cells were then permeabilized in 0.2% Triton solution and co-stained with rabbit anti-FAT10 polyclonal antibody (Ren et al., 2006) and mouse anti-p65 monoclonal IgG (Santa Cruz). Alexa Fluor 647 chicken anti-mouse or Alexa Fluor 488 anti-rabbit IgG (Molecular Probes, Inc., USA) were used as secondary antibodies. Cells were also incubated with DAPI to stain cell nucleus. Cellular localization observations were performed on the LSM510 confocal microscope (Carl Zeiss, Germany).

## Supplementary Material

Supplementary Material
