# Supplementary material for: Silibinin down-regulates FAT10 and modulate TNF-α/IFN-γ-induced chromosomal instability and apoptosis sensitivity
Source: Biol Open. 2015 Jul 3;4(8):961–9. doi: 10.1242/bio.011189 (PMC4542280; doi:10.1242/bio.011189)
Supplement: Supplementary Material [file supp_4_8_961__index.html]

Silibinin down-regulates FAT10 and modulate TNF-α/IFN-γ-induced chromosomal instability and apoptosis sensitivity — Silibinin down-regulates FAT10 and modulate TNF-α/IFN-γ-induced chromosomal instability and apoptosis sensitivity — Supplementary Material 

# Silibinin down-regulates FAT10 and modulate TNF-α/IFN-γ-induced chromosomal instability and apoptosis sensitivity

## BIO011189 Supplementary Material

- Supplementary Material
